# A Novel Graphene Oxide/γ-Alumina Hybrid Nano-Adsorbent Integrated into Polyurethane Foam for Adsorption of Industrial Pollutants from Wastewater

**DOI:** 10.3390/nano16191239

**Published:** 2026-09-30

**Authors:** Salar Sadri, Mohammad Hassan Vakili, Nader Mokhtarian

**Affiliations:** Department of Chemical Engineering, Shah.C, Islamic Azad University, Shahreza 8648146411, Iran; salar.sadri@gmail.com (S.S.); nmokhtarian@gmail.com (N.M.)

**Keywords:** graphene oxide (GO), γ-alumina nanoparticles, hybrid nanocomposites, wastewater treatment, heavy metal adsorption

## Abstract

This study reports the synthesis, characterization, and wastewater treatment performance of a hybrid nanocomposite based on graphene oxide (GO) and γ-alumina (γ-Al_2_O_3_). Functionalization was carried out via silanization using GPTMS for GO and APTES for γ-Al_2_O_3_, followed by covalent grafting through epoxy–amine linkage. FTIR spectroscopy confirmed successful grafting, showing Si–O–Si stretching and reduced epoxy ring vibrations. BET analysis indicated that the GO/γ-Al_2_O_3_ hybrid (ALGO) preserved a high specific surface area (198.08 m^2^/g), comparable to commercial γ-Al_2_O_3_, demonstrating that GO did not hinder porosity. ALGO was incorporated into polyurethane foam at loadings of 1–5% (ALGO@PU foam) and applied in wastewater treatment. UV-Vis spectroscopy revealed strong affinity for silver ions, evidenced by a decrease in AgNO_3_ absorbance (~430–600 nm) with increasing AlGO content. Treatment of real industrial wastewater showed significant reductions in chemical oxygen demand (COD), biological oxygen demand (BOD), and turbidity. At 5% loading, COD and BOD decreased from 1162 and 480 mg/L to 64 and 11 mg/L, respectively, while turbidity dropped from >1100 NTU to 1.62 NTU. These results demonstrate that ALGO is a functional adsorbent capable of removing ion metals, and improving water clarity, highlighting its potential for industrial wastewater treatment applications.

## 1. Introduction

Water pollution caused by heavy metals and organic dyes poses a critical threat to ecosystems and human health worldwide. Industrial effluents from mining, electroplating, textile, and paint industries discharge toxic ions such as Pb^2+^, Cd^2+^, and Cr^6+^, alongside organic pigments and dyes, into water bodies, leading to bioaccumulation in aquatic organisms and potential carcinogenic effects in humans [1,2]. Conventional treatment methods—chemical precipitation, ion exchange, membrane filtration, and biological processes—often face challenges of high cost, secondary pollution, low selectivity, and limited reuse of sorbents [3].

Adsorption has emerged as a superior technique due to its operational simplicity, cost effectiveness, and adaptability to various contaminants. Recent research has focused on developing novel adsorbent materials with high surface area, tunable surface chemistry, and mechanical robustness. Among these, graphene oxide (GO) stands out owing to its two-dimensional structure, abundant oxygenated functional groups (epoxy, hydroxyl, carboxyl), and exceptionally large specific surface area (~2630 m^2^/g), facilitating rapid and high-capacity adsorption of both heavy metal ions and organic dyes [1,2,3,4]. GO’s surface charge and functional group density can be tailored via oxidation degree and post-functionalization, enabling selective interactions with cationic or anionic pollutants [4,5].

Gamma-phase alumina (γ-Al_2_O_3_) nanoparticles have also been widely employed as adsorbents for heavy metals, owing to their high surface area, thermal stability, and abundant hydroxyl surface sites that complex with metal ions [6,7]. However, standalone γ-Al_2_O_3_ suffers from limited adsorption of organic dyes and tends to agglomerate in aqueous media, reducing effective surface area [8]. To overcome these drawbacks, hybrid composites of GO and γ-Al_2_O_3_ have been explored, leveraging the synergistic combination of GO’s versatile functional groups and γ-Al_2_O_3_’s strong affinity for metal ions. These hybrids exhibit enhanced pollutant removal performance, improved stability, and tunable surface properties [8,9].

Supporting adsorptive nanoparticles on three-dimensional (3D) macroporous substrates addresses issues of nanoparticle recovery, pressure drop, and mechanical handling in fixed-bed or column operations. Polyurethane (PU) foam is a promising scaffold due to its low density, open-cell structure, mechanical flexibility, and ease of functionalization [10,11,12]. PU foam can be impregnated with nanoparticles via dip coating, in situ synthesis, or dynamic covalent bonding, creating robust nanocomposite foams with uniformly distributed active sites [13].

Graphene-based PU foams have been demonstrated for various applications, including EMI shielding, thermal insulation, and oil/water separation [14]. Incorporation of metal oxide nanoparticles—such as TiO_2_, ZnO, or γ-Al_2_O_3_—into PU/GO foams further endows additional adsorption or photocatalytic functionalities [15,16,17]. For instance, a GO/TiO_2_/PU foam achieved over 95% removal of methyl orange under UV irradiation, while maintaining structural integrity over multiple cycles [18].

Despite these advances, most hybrid PU foams target either heavy metals or organic dyes separately. Simultaneous removal of multi-contaminant streams remains a significant challenge, as optimal conditions (pH, ionic strength, competing ions) for one pollutant class may differ from those for another [19,20,21]. Furthermore, few studies have systematically tailored the ratio of GO to γ-Al_2_O_3_, the degree of surface oxidation, and the binding chemistry to maximize dual-mode adsorption performance.

To address these gaps, recent work has explored covalent grafting of PU hard segments onto GO to enhance nanoparticle anchoring and prevent leaching [21]. Others have investigated polymer nanocomposites infused with metal oxide fillers for broad-spectrum adsorption of heavy metals, dyes, and organic micropollutants [22]. Yet, a comprehensive design of a GO/γ-Al_2_O_3_ hybrid supported on PU foam, optimized for simultaneous heavy metal and organic dye removal under realistic wastewater conditions, is still lacking.

In this study, we report the fabrication and characterization of a novel GO/γ-Al_2_O_3_ (ALGO) nanocomposite immobilized on PU foam for the efficient and simultaneous separation of Ag^+^ from aqueous solutions. The hybrid foam was synthesized via a facile dip coating approach, followed by thermal curing to covalently anchor γ-Al_2_O_3_ nanoparticles onto GO sheets and subsequently embed them within the PU scaffold [23,24,25,26]. We investigate the effects of ALGO ratio in PU foam for ion adsorption. This work provides a promising strategy for cost-effective, scalable wastewater treatment using multifunctional PU-supported nanocomposites.

## 2. Experimental Section

### 2.1. Materials

Commercial open-cell polyurethane foam with a density of 30 kg/m^3^ was purchased from Foamex Inc. and cut into pieces measuring 2 cm × 2 cm × 1 cm. Analytical grade hexahydrate aluminum chloride (AlCl_3_·6H_2_O) was obtained from Sigma-Aldrich (St. Louis, MO, USA). Graphene oxide (GO) with a lateral size ranging from 0.5 to 5 μm and a specific surface area of 350 m^2^/g was sourced from Nanotech (Tehran, Iran). (3-Glycidyloxypropyl) trimethoxysilane (GPTMS) with a purity of 97% was acquired from Gelest, and (3-aminopropyl) trimethoxysilane (APTES) with a purity of 99% was obtained from Sigma-Aldrich. Ethanol and toluene were procured from Merck (Darmstadt, Germany).

### 2.2. Synthesis of γ-Al_2_O_3_ Nanoparticles

A 0.5 M solution of aluminum chloride was prepared in 200 mL of deionized water and heated to 60 °C under continuous stirring. Aqueous ammonia (25 wt%) was added dropwise until the pH of the solution reached 8.5–9.0, forming a gelatinous precipitate of aluminum hydroxide. The suspension was aged at 60 °C for 2 h to encourage crystallization of the hydroxide phase. The resulting precipitate was separated via centrifugation at 10,000 rpm for 10 min, washed several times with deionized water until neutral pH was reached, and then dried overnight at 100 °C. Finally, the dried precursor was calcined in a muffle furnace by ramping the temperature to 500 °C at a rate of 5 °C min^−1^ and holding it for 4 h, yielding γ-Al_2_O_3_ nanoparticles (SAl).

### 2.3. Functionalization of GO with GPTMS (GG Nanoparticles)

Graphene oxide (GO) was functionalized using GPTMS to introduce epoxy functionalities for subsequent grafting. In this process, 200 mg of GO was first dispersed in a 1:1 mixture of ethanol and deionized water (100 mL total) using ultrasonication for 30 min to ensure uniform suspension. Subsequently, 2 mL of GPTMS and 1 mL of water were added to the suspension. The pH was adjusted to approximately 4 using glacial acetic acid to promote hydrolysis of the alkoxysilane groups into reactive silanols. The mixture was stirred at 60 °C for 6 h to allow silanization to proceed efficiently. The resulting silanized GO was separated via centrifugation, washed thoroughly with ethanol to remove unreacted GPTMS and byproducts, and dried at 60 °C to yield GG.

### 2.4. Functionalization of γ-Al_2_O_3_ with APTES (As Nanoparticles)

For the functionalization of γ-Al_2_O_3_ nanoparticles, 200 mg of the synthesized alumina was dispersed in a 1:1 ethanol–deionized water mixture (100 mL) using ultrasonication for 30 min to achieve uniform dispersion. To this suspension, 2 mL of APTES and 1 mL of water were added. The pH was then adjusted to ~4 using acetic acid to facilitate hydrolysis of the ethoxysilane groups in APTES to form silanol species. The reaction was carried out under continuous stirring at 60 °C for 5 h to enable covalent bonding of the silanols to the alumina surface. Following completion, the APTES-modified γ-Al_2_O_3_ (AS) was recovered by centrifugation, thoroughly washed with ethanol, and dried at 80 °C.

### 2.5. Grafting of Functionalized γ-Al_2_O_3_ onto Functionalized GO (Preparation of ALGO Nanoparticle)

**Scheme 1:**
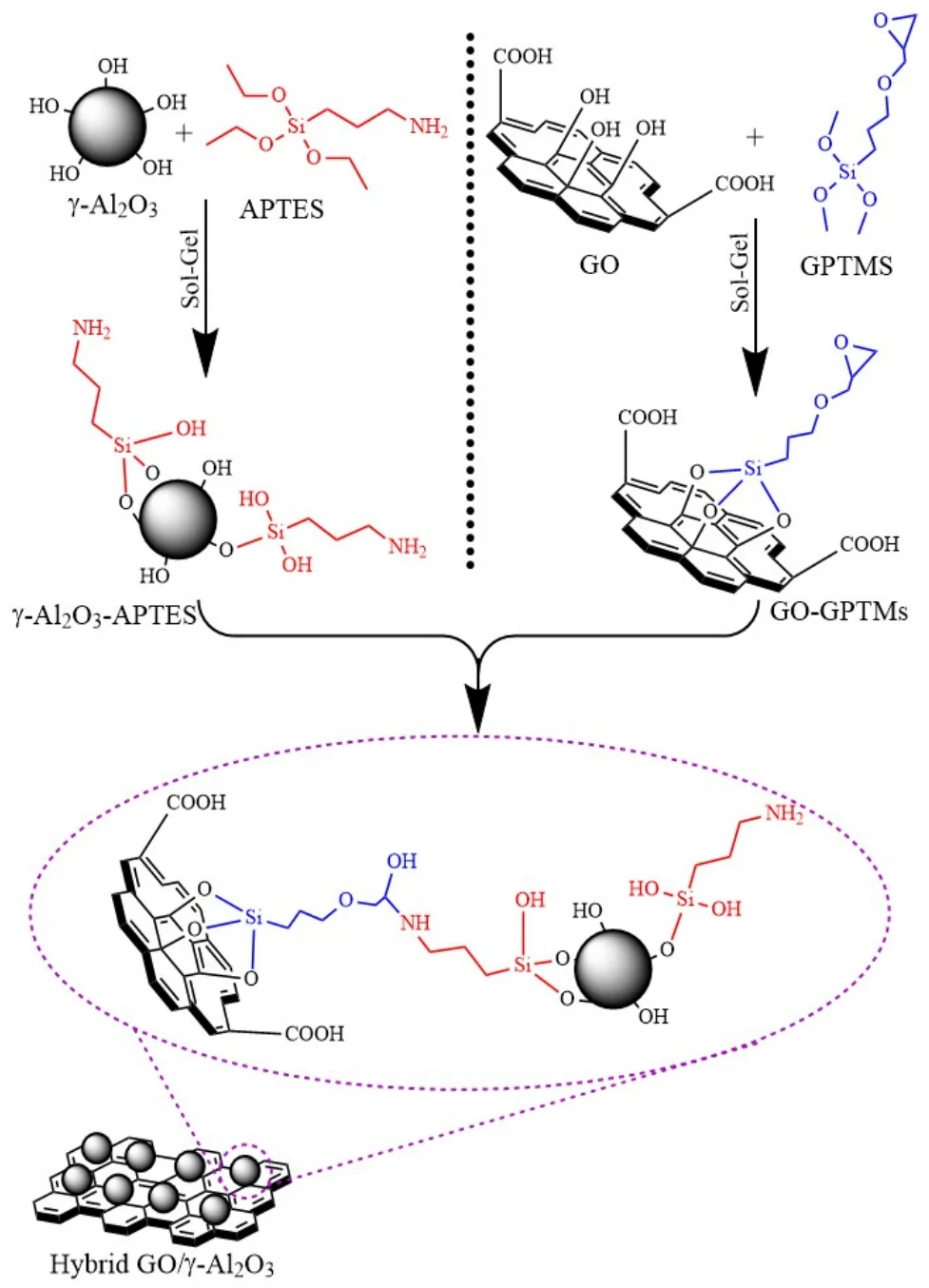
The chemical modification of alumina, graphene, and their hybrid synthesis.

The covalent grafting of APTES-functionalized γ-Al_2_O_3_ onto GPTMS-functionalized GO was carried out in anhydrous toluene to promote the reaction between epoxy and amine groups on the respective silane-modified surfaces. In a typical procedure, 100 mg of GG and 100 mg of AS were dispersed together in 80 mL of anhydrous toluene under ultrasonication for 30 min to ensure homogeneous mixing. The suspension was then refluxed at 110 °C under nitrogen atmosphere for 24 h with continuous stirring to allow the epoxy groups of GPTMS to react with the amine groups of APTES, forming covalent C–N linkages. After cooling to room temperature, the AlGO nanoparticles were collected by centrifugation, washed several times with toluene and ethanol to remove unreacted species, and dried under vacuum at 60 °C. The schematic of the reactions is shown in Scheme 1.

### 2.6. Preparation of ALGO@PU Nanocomposite Foams

The polyurethane foam was fabricated following the manufacturer’s protocol, which specifies a 1:1 weight ratio of polyol to isocyanate. For the preparation of the ALGO@PU nanocomposite foam, 1–5 wt% of the hybrid nanoparticles (relative to the total foam weight) was first dispersed into the polyol using mechanical stirring at 2000 rpm for 30 min to ensure uniform distribution. The isocyanate component was then added during mixing, and the blend was stirred for an additional 2 min, after which foam formation occurred. The resulting samples were allowed to cure for 24 h before being subjected to UV–Vis, BOD, COD, and turbidity analyses.

### 2.7. Characterization

- **Biochemical Oxygen Demand (BOD) and Chemical Oxygen Demand (COD):** These are standard methods (APHA 5210 B and 5220 D) to assess organic load before and after treatment.
- **Turbidity:** This was measured with a Hach 2100N turbidimeter (NTU scale) to monitor colloidal removal efficiency.
- **Fourier-Transform Infrared Spectroscopy (FTIR):** Nicolet iS50 FTIR in the 4000–400 cm^−1^ region (KBr pellet) to confirm functionalization and grafting.
- **UV–Vis Absorption:** Shimadzu UV-2600 spectrophotometer to quantify ion concentration.
- **Brunauer–Emmett–Teller (BET) Surface Area and Porosity:** Micromeritics ASAP 2020 analyzer: N_2_ adsorption–desorption isotherms at 77 K to determine specific surface area, pore volume, and pore size distribution.
- **Raman analysis:** The order/disorder of GO and AlGO and any structural changes according to modification were found by Raman spectroscopy, which was carried out on a Raman spectrometer (Takram P50C0R10 model Teksan, laser excitation wavelength = 514 nm).
- **XRD:** X-ray diffraction (XRD) was utilized to investigate the crystalline structures formed on the surface of graphene. The patterns obtained were measured using a D5000 X-ray diffractometer (Siemens, Germany) equipped with Cu Kα radiation (0.1540 nm).
- **FESEM:** Field emission scanning electron microscopy was used to examine the surface morphology of the graphene and evaluate the success of the final synthesis. Images were acquired using a MIRA3 TESCAN XMU instrument.
- **TEM:** Transmission electron microscopy (TEM) was performed using a Philips EM 208 (The Netherlands) operating at an accelerating voltage of 80 kV. The samples were prepared by depositing a thin layer onto lacy grids.

## 3. Result and Discussion

### 3.1. FTIR Analysis

**Figure 1:**
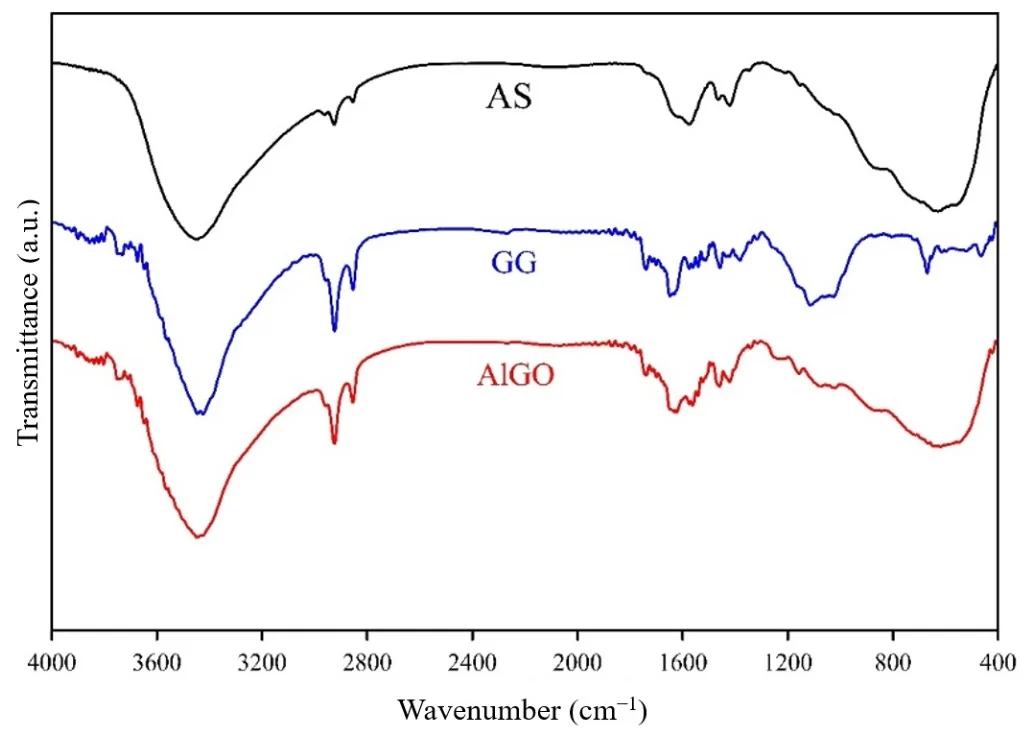
FTIR spectra of GPTMS-functionalized GO (GG), APTES-functionalized γ-alumina (AS), and hybrid GO/γ-alumina nanoparticles (ALGO).

Fourier-transform infrared (FTIR) spectroscopy was used to verify the surface functionalization of graphene oxide (GO) with 3-glycidyloxypropyltrimethoxysilane (GPTMS), γ-alumina with 3 aminopropyltriethoxysilane (APTES), and their covalent integration into a hybrid GO/γ-alumina nanostructure. The corresponding spectra of silanized GO (GG), silanized γ-alumina (AS), and the hybrid composite (ALGO) are presented in Figure 1. In the GG spectrum, the peak near 1100 cm^−1^ is attributed to asymmetric Si–O–Si stretching, confirming the successful condensation of GPTMS on the GO surface through siloxane network formation [27,28]. A peak at ~2925 cm^−1^ corresponds to the C–H stretching vibrations of the GPTMS propyl chains. The broad band around 3400 cm^−1^ represents O–H stretching from hydroxyl or silanol groups. The presence of a weak band at ~910 cm^−1^, characteristic of the epoxy ring of GPTMS, confirms the preservation of reactive epoxide groups [27,28]. The AS spectrum of APTES-functionalized γ-alumina also shows a Si–O–Si band near 1100 cm^−1^, along with a C–H stretching peak at ~2925 cm^−1^. A peak around 1570 cm^−1^ is attributed to N–H bending of primary amines introduced by APTES [29,30]. The broad band at ~3400 cm^−1^ reflects overlapping N–H and O–H stretching vibrations, typical for amine-functionalized surfaces [30]. In the ALGO hybrid spectrum, the persistence of Si–O–Si and alkyl stretching bands confirms the presence of both GPTMS and APTES. Importantly, the epoxy peak at ~910 cm^−1^ observed in GG is significantly reduced or absent in ALGO, indicating the consumption of epoxy groups. Concurrently, the N–H bending peak (~1570 cm^−1^) shows a shift and change in intensity, consistent with the reaction between amine groups and epoxy rings [28,30]. These observations suggest that the amine groups of APTES underwent a nucleophilic ring-opening reaction with the epoxide of GPTMS, forming covalent β-amino alcohol linkages, thus grafting GO and γ-alumina into a hybrid structure [30]. These spectral features validate the chemical pathways proposed for silanization and confirm the covalent integration between the two nanomaterials.

### 3.2. BET Surface Area Analysis

Brunauer–Emmett–Teller (BET) surface area measurements were conducted to evaluate the specific surface area and porosity characteristics of synthesized alumina (SAl), commercial γ-alumina (AL), and the ALGO. The results are compiled in Table 1. The synthesized alumina (SAl) exhibited a specific surface area of 117.26 m^2^/g, which is significantly lower than that of commercial γ-alumina (AL), measured at 200.03 m^2^/g. This difference can be attributed to variations in synthesis conditions, particle morphology, and thermal treatment, which strongly influence surface structure and pore accessibility [31]. The ALGO demonstrated a specific surface area of 198.08 m^2^/g, closely comparable to that of the commercial γ-alumina. This observation suggests that the incorporation of graphene oxide into γ-alumina did not significantly obstruct pore accessibility or cause agglomeration. In fact, it may enhance dispersion and create mesoporous frameworks that preserve high surface areas [32]. Furthermore, the slight reduction in surface area in ALGO compared to pure AL may be attributed to partial surface coverage of γ-alumina by graphene oxide sheets or the formation of interfacial linkages that reduce free surface exposure [33].

### 3.3. UV-Vis Absorption Analysis

**Figure 2:**
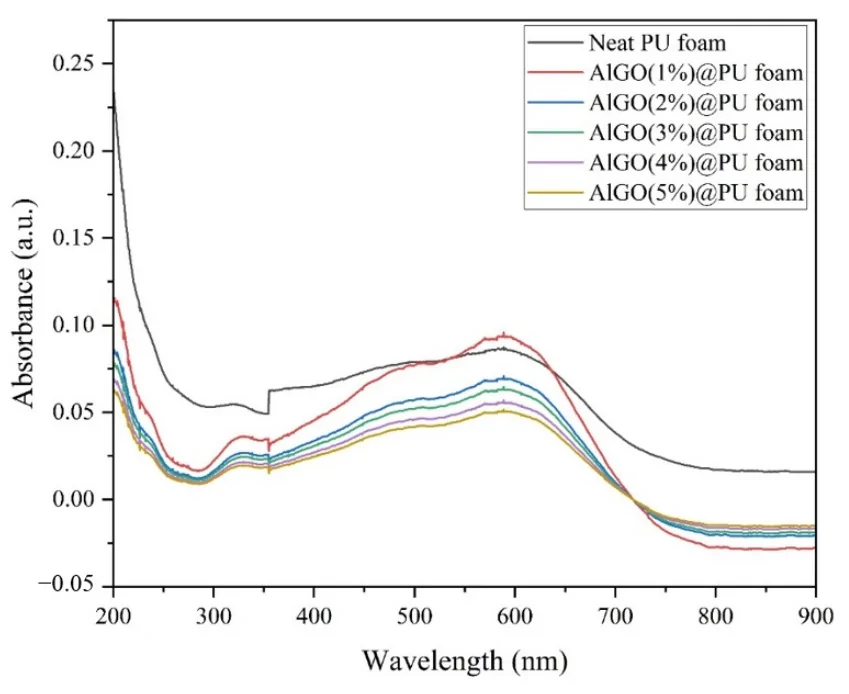
UV–Visible absorption spectra of AgNO_3_ solution in the presence of different loadings (1–5 wt%) of ALGO@PU foam.

UV–Visible spectroscopy was conducted to evaluate the ability of ALGO@PU nanocomposite foams to reduce silver nitrate (AgNO_3_). The samples analyzed contained increasing concentrations of the hybrid nanocomposites (1–5 wt%), and their influence on the optical absorbance of Ag was recorded in the range of 200–900 nm. The results are presented in Figure 2. The control sample containing neat AgNO_3_ exhibited a strong absorption peak near 430–450 and 600 nm, characteristic of the surface plasmon resonance (SPR) of Ag in solution [34,35]. Upon the addition of ALGO@PU nanocompsite foam, a decrease in the intensity of this absorption band was observed as the nanoparticle concentration increased from 1% to 5%. This decrease in absorbance suggests the effective interaction between Ag and the surface functionalities of the ALGO nanostructure. GO contributes active oxygenated groups (epoxy, hydroxyl, and carboxyl), while γ-Al_2_O_3_ provides surface hydroxyls and high surface area. These features likely promote adsorption of Ag on the nanoparticle surface [36]. The decrease in intensity reflects the removal of Ag, possibly through surface interactions and physical adsorption. Interestingly, ALGO(5%)@/PU foam nanocomposites resulted in the lowest absorbance, indicating optimal performance at higher nanoparticle loading. This trend confirms a dose-dependent enhancement in silver-ion removal, aligned with the increase in active surface sites provided by the hybrid material [37].

### 3.4. Wastewater Treatment Performance

The turbidity, biological oxygen demand (BOD), and chemical oxygen demand (COD) of metal industry wastewater were measured after treatment with increasing concentrations (0%, 3%, 4%, 5%) of synthesized ALGO@PU foam nanocomposites (In UV-Vis analysis, it was determined that the 1% and 2% loadings did not demonstrate significant effectiveness; consequently, they were excluded from further analysis.). The corresponding values are summarized in Table 2. As seen in the results, the untreated (neat) sample showed high values of COD (1162 mg/L), BOD (480 mg/L), and turbidity (>1100 NTU), reflecting severe organic and particulate contamination. Upon treatment with ALGO(3%)@PU foam, there was a dramatic reduction in all three parameters, with COD decreasing to 65 mg/L, BOD to 14 mg/L, and turbidity to 3.60 NTU. A further decrease was observed with 4% and 5% loadings, where COD dropped to 64 mg/L and 64 mg/L, BOD to 13 mg/L and 11 mg/L, and turbidity to 3.29 NTU and 1.62 NTU, respectively. Although this reduction is not significant, the 3% loading yielded the best result. This performance enhancement is consistent with the UV–Vis results (Figure 2), which showed increasing reduction in AgNO_3_ absorbance with higher hybrid concentrations. The hybrid AlGO provides a synergistic effect: graphene oxide contributes high adsorption capacity via π–π interactions and surface functional groups, while γ-alumina enhances catalytic and adsorptive removal through its high surface area and surface hydroxyls [27,38,39]. The simultaneous removal of silver ions (as inferred from UV–Vis) and reduction in organic and particulate matter demonstrates that the AlGO hybrid acts as a multifunctional treatment agent. Its ability to reduce both BOD and COD reflects improved oxidative and adsorptive removal of pollutants—potentially due to the nanocomposite’s role in facilitating electron transfer and degrading or immobilizing organic contaminants [38].

### 3.5. XRD Analysis

**Figure 3:**
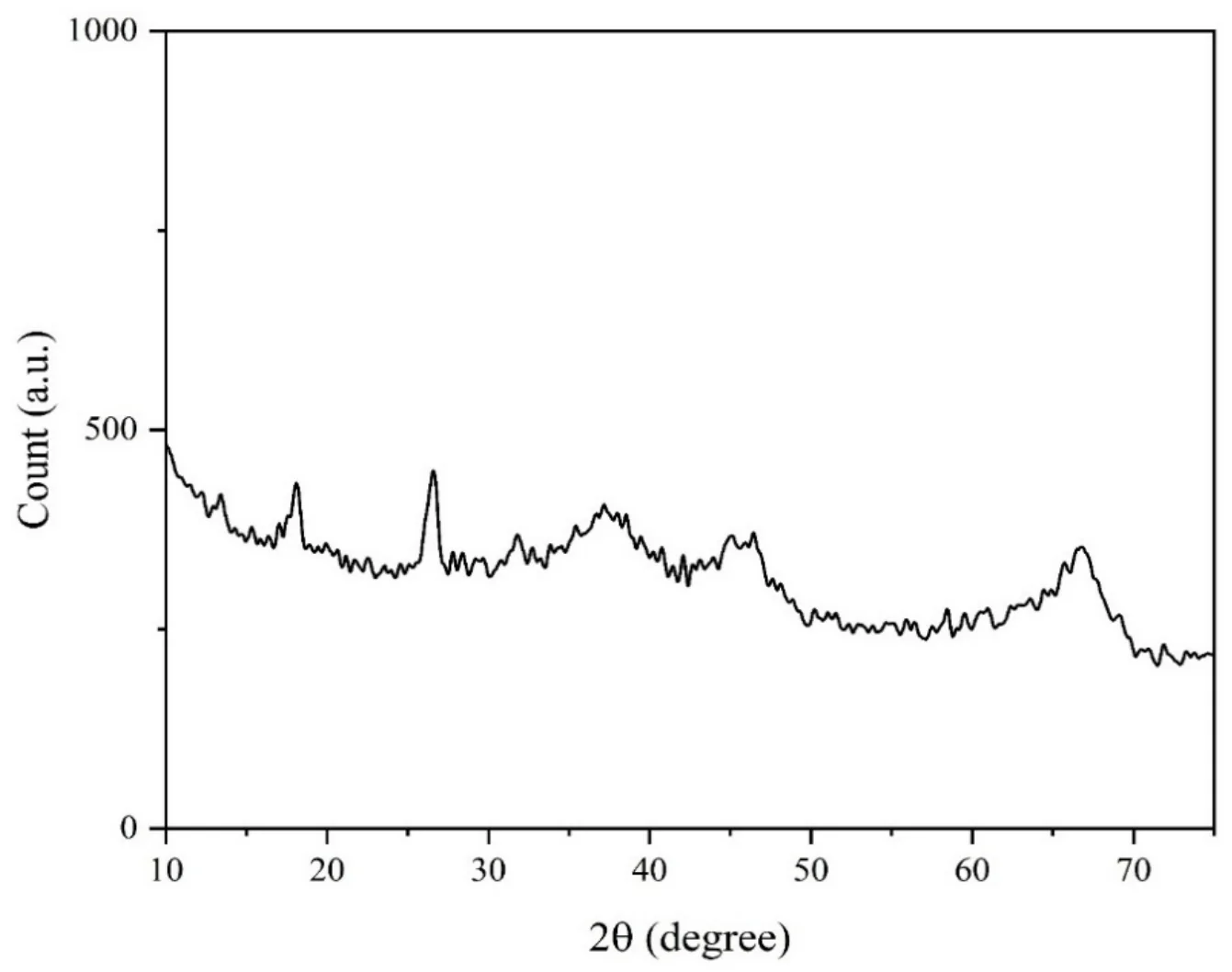
The XRD pattern of the ALGO hybrid nanoparticle.

The X-ray diffraction (XRD) analysis of the graphene–alumina hybrid nanoparticles reveals a pattern consistent with the successful synthesis of a nanostructured composite material (Figure 3). The prominent peak at approximately 26.6° (d-spacing ≈ 3.35 Å) corresponds to the (002) plane of GO, indicating the presence of graphene sheets with a broadened profile due to nanoscale disorder and stacking irregularities typical of hybrid materials. This absence of a peak around 11° (characteristic of graphene oxide) suggests effective exfoliation of nanolayers during synthesis, a critical step for enhancing the electrical and mechanical properties of the hybrid. Additionally, the low-angle high-intensity tail observed from 4° to 10° may be attributed to instrumental artifacts or large-scale structural features, though it does not detract from the identification of the graphene component [27,40].

Further examination of the XRD pattern identifies several broad peaks aligning with the gamma-phase alumina (γ-Al_2_O_3_), including those at 37.2° (d-spacing ≈ 2.42 Å, (311) plane), 46.4° (d-spacing ≈ 1.96 Å, (400) plane), and 67.1° (d-spacing ≈ 1.39 Å, (440) plane), confirming the incorporation of alumina nanoparticles. The broad peak widths (FWHM ~0.5–2°) suggest crystallite sizes in the range of 5–20 nm, consistent with the nanoscale nature of the hybrid, where alumina particles are likely anchored onto the graphene surface. Slight shifts in peak positions compared to pure γ-Al_2_O_3_ standards may indicate lattice strain or interfacial interactions between the graphene and alumina phases, a hallmark of successful hybridization. The absence of sharp peaks or additional phases (e.g., alpha-alumina or impurities) supports the purity and structural integrity of the synthesized material, making it suitable for applications such as catalysis, thermal management, or composite reinforcement [27,40].

### 3.6. Raman Analysis

**Figure 4:**
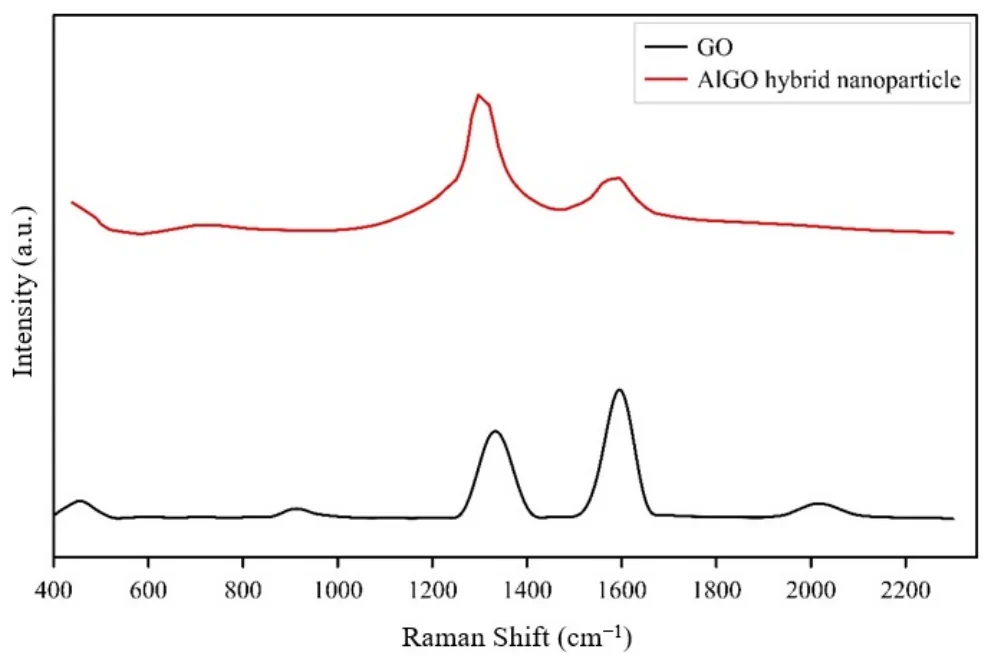
Raman spectra of GO and the ALGO hybrid.

The Raman spectra of GO and the ALGO hybrid are shown in Figure 4. The Raman spectrum of neat graphene oxide (GO) exhibits the well-known graphitic bands, with the defect-induced D band observed at approximately 1332 cm^−1^ and the G band at 1600 cm^−1^. The relative intensity ratio (ID/IG ≈ 0.6–0.7) indicates a moderate degree of structural disorder, which is consistent with the presence of oxygen functionalities introduced during the oxidation of graphite. Although GO contains defects, the relatively low I_D_/I_G_ value compared with heavily functionalized derivatives suggests that extended sp^2^ domains are still present, preserving partial conjugation and graphitic ordering. These features align with previously reported Raman signatures of GO, which display the coexistence of oxidized sp^3^ carbons and intact aromatic regions [41].

Upon grafting with silanes and anchoring onto alumina, notable changes arise in the Raman profile. The D band shifts to ~1307 cm^−1^ while the G band appears downshifted at ~1575 cm^−1^, with a marked increase in I_D_/I_G_ to approximately 2.1. Such an increase in defect-related intensity is attributed to the introduction of new sp^3^ centers as a result of covalent bonding between GPTMS and the GO basal planes. Furthermore, the downshift and broadening of the G band signify lattice distortion and possible charge-transfer interactions with the alumina substrate, mediated by APTES linkages. These modifications clearly demonstrate that the grafting process increases the defect density and reduces the average size of sp^2^ domains, confirming successful covalent functionalization and interfacial bonding. In addition to changes in the carbon framework, the grafted spectrum reveals new vibrational modes absent in neat GO. The s band at ~442 cm^−1^ corresponds to Al–O stretching from the alumina support. This result provides compelling evidence of a strongly bonded GO–alumina hybrid structure. This comparison highlights how Raman spectroscopy can effectively track both the structural disordering of graphene-based phases and the emergence of new interfacial linkages that validate the success of surface functionalization [41,42].

### 3.7. FESEM Images

**Figure 5:**
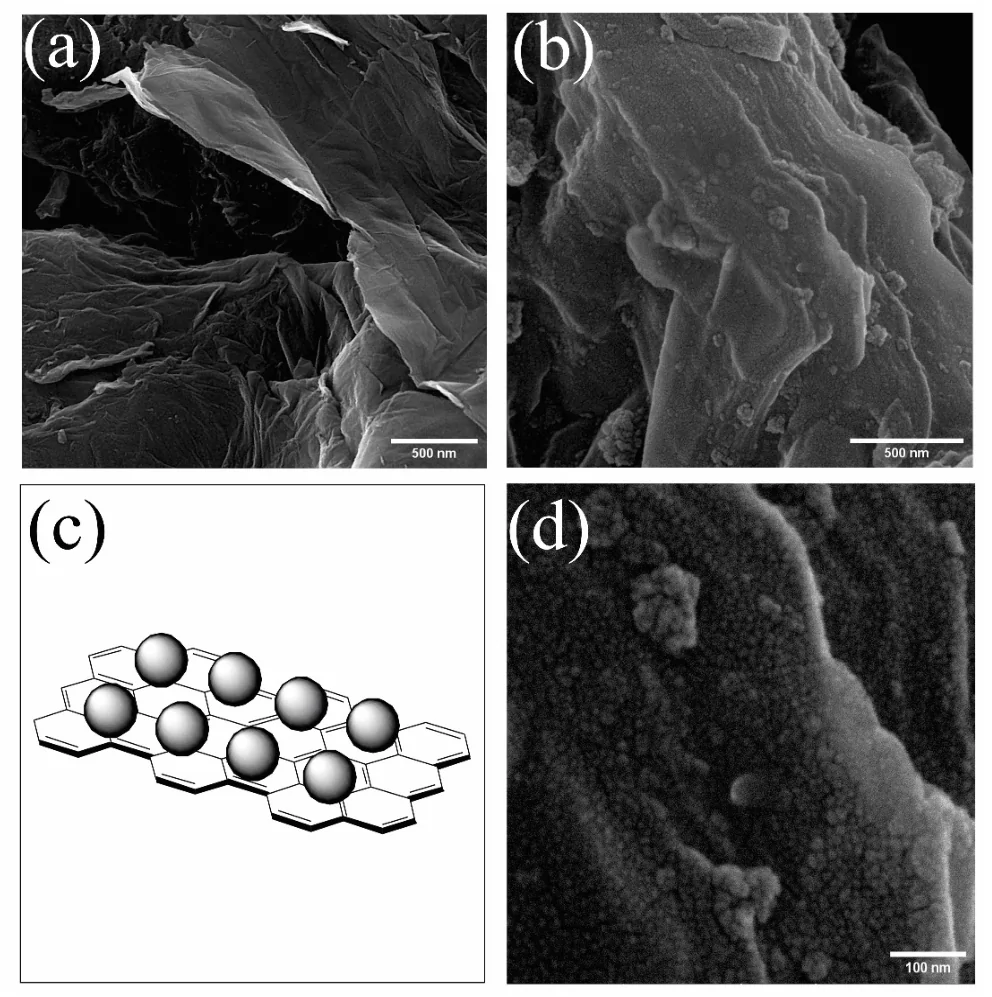
FESEM images of (**a**) GO and (**b**) ALGO; (**c**) schematic of nanoparticles are placed on the GO surface; (**d**) FESEM image of ALGO at higher magnification.

FESEM images of the GO and AlGO samples are presented in Figure 5. These images were acquired from thin films deposited onto mica substrates by spin-coating. Figure 5a shows the GO surface, which exhibits a wavy morphology characterized by distinct wrinkles interspersed with smooth regions. In contrast, Figure 5b displays the morphology after grafting of γ-Al_2_O_3_ onto the GO surface, where thickening of the sheets and increased surface roughness are clearly evident. At higher magnification (Figure 5d), spherical particles are distinctly visible on the GO surface, consistent with the grafting schematic illustrated in Figure 5c.

### 3.8. TEM Images

**Figure 6:**
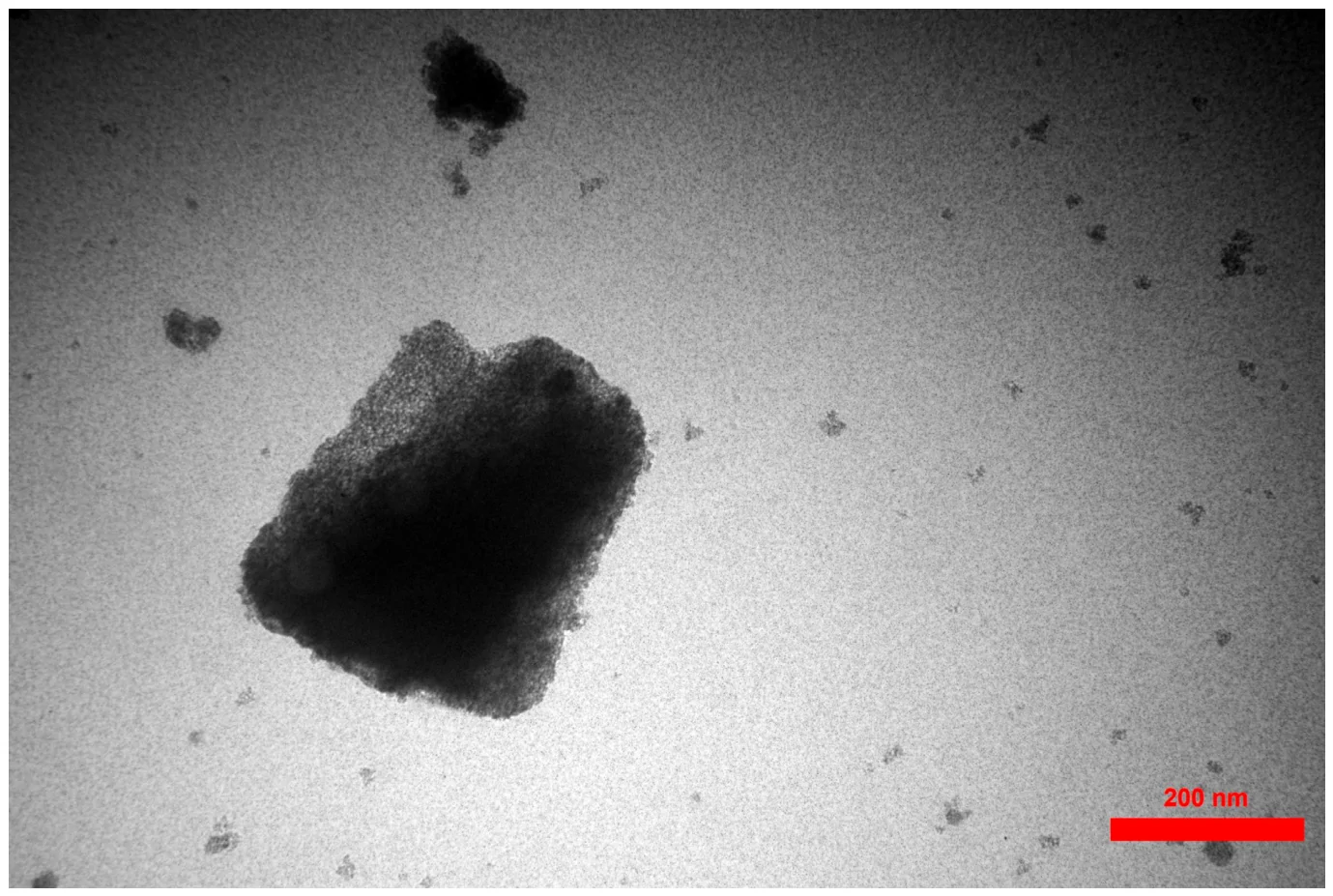
TEM images of the ALGO hybrid sample.

The TEM images with a 50 nm scale bar clearly reveal the successful formation of alumina–graphene hybrid nanostructures (Figure 6). The darker, spherical alumina nanoparticles are observed to be homogeneously distributed across the lighter graphene sheets. Their preferential attachment along the wrinkled surfaces and sheet edges suggests that the functional groups on graphene provided active sites for anchoring. Closer inspection highlights the strong interaction between alumina nanoparticles and graphene, where the particles are not merely physisorbed but appear embedded or “pinned” into the graphene substrate. The intimate interfacial contact, with no visible voids or detachment zones, supports the successful covalent bonding facilitated by amine and epoxy silane linkers. This covalent grafting ensures structural stability, leading to hybrid architectures that are robust against detachment or phase separation, a common limitation in physically mixed systems. The TEM results therefore provide direct visual confirmation of the designed hybridization strategy, validating that alumina nanoparticles are successfully grafted onto graphene sheets with nanoscale precision and structural integrity.

## 4. Conclusions

The study successfully developed a graphene oxide/γ-alumina (ALGO) hybrid nanocomposite for industrial wastewater treatment through covalent grafting using GPTMS and APTES silane agents. FTIR and BET analyses confirmed robust chemical integration, structural stability, and a high surface area (198.08 m^2^/g), promoting rapid adsorption. The ALGO@PU foam nanocomposite was prepared by loading 1 to 5% of ALGO into polyurethane foam and it was used for its performance in removing water pollutants by UV-Vis, BOD, COD and turbidity tests. UV-Vis tests indicated excellent silver-ion removal due to synergistic interactions from GO’s π-π stacking and γ-Al_2_O_3_’s surface chemistry. Real wastewater tests showed dramatic reductions in COD, BOD, and turbidity even at low nanoparticle loadings. The best results were obtained for ALGO(5%)@PU foam, which shows that this nanocomposite has structural stability, reduced secondary pollution risk, and good potential for reusability. Its facile, environmentally friendly synthesis allows adaptability to membrane or foam substrates. This nanocomposite addresses diverse pollutants simultaneously, providing a versatile, scalable, and sustainable platform for advanced wastewater purification technologies.

## Figures and Tables

**Table 1:** BET surface area and related adsorption properties of alumina samples and their graphene-based hybrid.

Samples	Adsorbed Volume (VSTP) [cc]	Monolayer Uptake Volume [cc]	Total Surface Area [m^2^]	Specific Surface Area [m^2^/g]
Synthesized Alumina (SAl)	0.51	0.36	1.5596	117.26
Commercial γ-Alumina (AL)	0.88	0.62	2.7004	200.03
ALGO	0.76	0.58	2.6024	198.08

**Table 2:** Effect of ALGO@PU foams on COD, BOD, and turbidity in treated wastewater.

Foam Nanocomposites	COD (mg/L)	BOD (mg/L)	Turbidity (NTU)
Neat PU foam	1162	480	>1100
ALGO(3%)@PU foam	65	14	3.60
ALGO(4%)@PU foam	65	13	3.29
ALGO(5%)@PU foam	64	11	1.62

## Data Availability

The original contributions presented in this study are included in the article. Further inquiries can be directed to the corresponding author.
